# Comprehensive Analysis Identified Glycosyltransferase Signature to Predict Glioma Prognosis and TAM Phenotype

**DOI:** 10.1155/2023/6082635

**Published:** 2023-01-11

**Authors:** Yiwei Qi, Wenchang Lv, Xiaojin Liu, Quanji Wang, Biao Xing, Qian Jiang, Zihan Wang, Yimin Huang, Kai Shu, Ting Lei

**Affiliations:** ^1^Department of Neurosurgery, Tongji Hospital, Tongji Medical College, Huazhong University of Science and Technology, Wuhan, China; ^2^Laboratory Sino-German Neuro-Oncology Molecular, Department of Neurosurgery, Tongji Hospital, Tongji Medical College, Huazhong University of Science and Technology, Wuhan, China; ^3^Department of Plastic and Cosmetic Surgery, Tongji Hospital, Tongji Medical College, Huazhong University of Science and Technology, Wuhan, China

## Abstract

Glycosylation is the most common posttranslational modification of proteins. Glycosyltransferase gene differential expression dictates the glycosylation model and is epigenetically regulating glioma progression and immunity. This study is aimed at identifying the glycosyltransferase gene signature to predict the prognosis and immune characteristics of glioma. The glycosyltransferase gene signature of glioma was identified in the TCGA database and validated in the CGGA database. Glioma patients were then divided into high- and low-risk groups based on risk scores to compare survival differences and predictive capacity. Subsequently, validation of glycosyltransferase gene signature merits by comparing with other signatures and utility in clinical judgment. The immune cell infiltration, immune pathways, and immune checkpoint expression level were also analyzed and compared in the high- and low-risk groups. Finally, the signature and its gene function were tested in our cohort and in vitro experiments. Eight glycosyltransferase genes were identified to establish the glycosyltransferase signature to predict the prognosis of glioma patients. The survival time was shorter in the high-risk group compared to the low-risk group based on glycosyltransferase signature and was confirmed in an independent external cohort. The glycosyltransferase signature displayed outstanding predictive capacity than other signatures in the TCGA and CGGA database cohorts. Furthermore, patients in the high-risk group were positively correlated with TAM infiltration, immune checkpoint expression level, and protumor immune pathways in TCGA cohorts. Validation of clinical tissue specimens revealed that the high-risk group was closely associated with infiltration of M2 TAMs. High-risk genes in the signature promote glioma proliferation, invasion, and macrophage recruitment in an in vitro validation of U87 and U251 cell lines. This carefully constructed that glycosyltransferase signature can predict the prognosis and immune profile of gliomas and help us evaluate subsequent macrophage-targeted therapies as well as other immune microenvironment modulation therapeutic strategies.

## 1. Introduction

Gliomas are the primary neoplasms found in the central nervous system (CNS) that arise from mutated neural stem cells or abnormal progenitor cells. Gliomas comprise about 30-50% of all human intracranial tumors, with an annual incidence rate estimated at 5 to 6 out of 100,000 [1]. Gliomas are usually associated with serious complications that severely reduce the quality of life and have fatal outcomes. The 2-year overall survival (OS) rate for newly diagnosed glioblastoma, even with radiation-based therapy combined with temozolomide, remains less than 27% [2]. Previously, gliomas were classified as astrocytomas, oligodendrogliomas, or ventricular meningiomas on the basis of histologic features and were categorized by the World Health Organization (WHO) as grades I-IV according to the tumor malignancy [3]. However, treatment based on histological classification alone did not dramatically prolong patient survival time. The landmark discovery of the isocitrate dehydrogenase (IDH) molecule pushes glioma treatment to new horizons [4]. Moreover, the latest WHO glioma classification increasingly highlights molecular features and reclassifies gliomas based on molecular features, indicating the role of characteristic molecular indicators in guiding glioma treatment [5]. Thus, the establishment of a valid molecular characterization model could benefit the treatment of glioma.

Glycosylation is the attachment of glycosyl groups to proteins and is a posttranslational modification (PTM) that provides proteomic diversity for cancer [6]. Altered glycosylation drives cancer development and progression as an important biomarker and provides a specific set of targets for therapeutic interventions, highlighting the role of glycan chains in cancer. The most widespread cancer-related glycosylation alterations include O-glycan truncation, sialylation, N-linked glycan branching, fucosylation, and O-linked glycan branching, which are associated with tumor invasion, proliferation, metastasis, and immunity [7]. It has been reported that induction of the glycosyltransferase 8 structural domain containing 1 (GLT8D1) by HIF-1*α* in a hypoxic environment is significantly associated with faster proliferation and poorer clinical outcomes in gliomas [8]. Peptide-O-linked mannose beta-1,2-N-acetylglucosaminyltransferase 1 (PomGnT1) regulates progression of glioma by activating the beta-catenin pathway and could work as a factor in the prognosis and treatment of glioma as well as a neotherapeutic molecular target. Glioma stem cell (GSC) invasion is associated with an altered N-glycosylation pattern. *α*-1,6-Mannosylglycoprotein 6-*β*-N-acetylglucosaminyltransferase (MGAT5) was reported to be a promoter of glioma invasion by catalyzing the expression of multibranched N-glycan chains [9]. Consequently, glycosylation is involved in the progression and development of gliomas.

Recently, it has been suggested that altered glycosylation is an important event in predicting tumor prognosis and immunotherapy response [10]. Almost all critical molecules involved in adaptive and innate immune responses are glycoproteins that can be modified by glycosylation. B3gnt2 modifies poly-N-acetyllactosamine synthase to disrupt tumor T cell interactions, reducing T cell activation [11]. On the other side, glioblastoma triggers inhibitory signaling in tumor-associated macrophages (TAMs) through local and distal immunoregulation by regulating the expression levels of O-linked glycans on the cell surface [12]. In addition, PD-L1 in cancer cells is normally frequently stabilized by modulating n-glycosylation to antagonize the binding of GSK3*β* [13]. Therefore, glycosylation is implicated in the regulation of the immune system and may predict glioma immunotherapy.

Glycosylation reactions, mostly catalyzed by glycosyltransferases (GTs), are almost ubiquitous in tumor evolution. Therefore, it is of great interest to explore the potential of GTs in the construction of prognostic predicting risk models for glioma. To address this issue, in the present study, we first filtered 8 differentially expressed GT genes for constructing a prognostic glycosyltransferase signature according to the genomic information of the TCGA database. Glioma cases were divided into low-risk and high-risk groups in accordance with their median risk scores. There were significant differences in gene expression level, OS, immune cell infiltration, and levels of immune checkpoint inhibitor between the high- and low-risk groups. We performed validation in both glioma samples and in vitro experiments to further confirm the accuracy of our glycosyltransferase signature for prognosis and evaluation of TAMs. This well-documented glycosyltransferase signature is an additional valuable predicative method for combating glioma.

## 2. Methods

### 2.1. Sources and Preprocessing of Datasets

Publicly available gene expression profiles and associated medical information were acquired from the Chinese Glioma Genome Atlas (CGGA) database and The Cancer Genome Atlas (TCGA) database. In our study, we excluded cases with no survival information. Finally, 668 glioma collected cases from the TCGA database were enrolled in the analysis. Meanwhile, the CGGA database containing 929 patients was used as the training set for analysis.

### 2.2. Gene Ontology and Kyoto Encyclopedia of Genes and Genomes Analyses

Enrichment of function and pathway of differentially expressed GT genes between glioma cases and normal samples was executed using the online database, including Gene Ontology (GO) pathway analysis and Kyoto Encyclopedia of Genes and Genomes (KEGG) pathway analysis, with the “enrichplot” R package [14]. The false discovery rate (FDR) with a setting of *p* < 0.05 and *q* < 0.05 was regarded as statistically significant after 1,000 permutations were conducted.

### 2.3. Construction and Validation of Glycosyltransferase Signature

A total of 169 GT genes were obtained through GlycoGene DataBase (GGDB). To find out the prognostic relevant GT genes, 169 GT genes belonging to the training cohort were first analyzed by univariate Cox regression to pick 149 GT genes in relation to glioma prognosis (*p* < 0.05). Glioma prognosis was analyzed by calculating the OS of glioma cases by the Cox regression analysis. To eliminate redundant GT genes for subsequent model development, LASSO regression was also performed. A glycosyltransferase signature predicting prognosis in glioma patients was developed utilizing multivariate Cox regression analysis. The risk score calculation was determined according to the formula as follows: Risk score = ∑_*i*=1_^*n*^(*βi*∗Expi). After yielding risk scores for all patients, patients were allocated to either the low-risk group or the high-risk group, based on the median risk score. The cases were classified in a two-dimensional scatter plot using principal component analysis (PCA). A validation set of CGGA was adopted to verify the universality of risk glycosyltransferase signature in glioma patients. The Kaplan-Meier (K-M) survival analysis by R program v4.03 was used to assess prognostic differences for the low-risk and high-risk groups. The survival prediction accuracy of the glycosyltransferase signature in 1, 3, and 5 years was validated through the receiver operating characteristic (ROC) curve.

### 2.4. Evaluating Signature Performance and Constructing Nomogram

Since glycosyltransferase signature and miscellaneous clinical factors, including age, grade, gender, Karnofsky performance scale (KPS), primary recurrent secondary (PRS) type, chemotherapy, and radiation therapy, were covariates, the prognosis value of glycosyltransferase signature characteristics was evaluated utilizing independent Cox regression analysis and multivariate Cox regression prognosis. Visualization of hazard ratios (HRs) and corresponding *p* values were presented in a forest plot. Subsequently, a nomogram was produced by the R “rms” package for assessing OS at 1, 3, and 5 years in glioma patients depending on the glycosyltransferase signature and associated clinical parameters. The nomogram is a visual representation of multivariate Cox regression and predicts OS in patients with glioma [15]. ROC curve analysis, concordance index (C-index), and calibration plots were employed to evaluate the predictive accuracy of the glycosyltransferase signature, elucidating the survival predictive value of glycosyltransferase signature in different clinical states by K-M survival analysis.

### 2.5. Immunologic Infiltration Analysis

22 immune infiltrated cells were analyzed in relative percentages with the “Cibersort” software package. Then, the fraction of 22 immune cell subpopulations was measured with the single-sample genomic enrichment analysis (ssGSEA), as well as 29 critical pathways in the samples (R “gsva” package). Subsequently, the ESTIMATE algorithm was carried out for calculating the stromal scores and immune scores as well as the tumor purity of the glioma samples. The “Reshape2” package was deployed to figure out the expression levels of immune checkpoints in both the high- and low-risk groups.

### 2.6. Clinical Data

The clinical follow-up data of the patients were obtained from the follow-up records of a retrospective clinical study in the Department of Neurosurgery, Tongji Hospital (TJIRB202111161). The information about patients was included in Table S1. All patients included in the study were treated with a standard surgical procedure and chemoradiotherapy and received regular postoperative follow-up and telephone follow-up. Patient tissue specimens were used for total RNA extraction and for histological evaluation. These specimens were ethically approved by Tongji Hospital (TJ IRB20211162).

### 2.7. Quantitative Real-Time PCR

Total RNA from glioma tissue was extracted utilizing the TRIzol kit (Servicebio, China). The NanoDrop 2000 spectrophotometer (Thermo Fisher Scientific, Waltham, MA, USA) was subsequently employed to estimate the purity and total RNA concentration. The cDNA synthesis kit (Yeasen, Shanghai, China) was applied to reverse transcribe RNA into complementary DNA (cDNA). The quantitative RT-PCR assays were performed on the PCR instrument (ABI Q1, CA, USA) with SYBR Green Master Mix (Yeasen, Shanghai, China) in triplicate samples. The primer sequences for RT-qPCR of these 8 GT genes are summarized in Table S2.

### 2.8. Immunohistochemistry and Immunofluorescence

First of all, tissue sections from glioma patients were subjected to deparaffinization, rehydration, and antigen retrieval. Anti-MGAT4B antibody (1 : 200, Abcam, San Diego, USA) was added to sections to interact with the antigen and then incubated at 4°C overnight. Next, the sections were washed and incubated with a secondary antibody conjugated to horseradish peroxidase (HRP) for about 1 h. Subsequently, a DAB peroxidase substrate kit was employed to perform the color development reaction (Maxin, Fuzhou, China). After fixation, the sections were finally photographed with a microscope (CKX53, Olympus Corporation, Tokyo, Japan).

For immunofluorescence (IF), tissue sections were also subjected to deparaffinization, rehydration, and antigen retrieval. Then, the anti-Iba1 antibody (1 : 200, Abcam, Sandiago, USA) was separately mixed with anti-MGAT4b antibody, anti-CD206 antibody (1 : 200, Proteintech, Chicago, USA), or anti-PD-L1 antibody (1 : 200, BioLegend, San Diego, USA). Subsequently, the sections were divided into different groups and incubated with the different mixed antibodies at 4°C overnight. After washing, the sections were incubated with DAPI and respectively fluorescently labeled second antibodies as required at room temperature for 2 h. After washing and fixation, pictures of immunofluorescence costaining were captured on a fluorescent microscope (CKX53, Olympus, Tokyo, Japan) and processed utilizing software ImageJ (Fiji version, NIH).

### 2.9. In Vitro Verification

The capability of glioma proliferation was detected with the Cell Counting Kit-8 (CCK-8) assay (Dojindo, Kumamoto, Japan). Human U87 and U251 glioma cell lines were used to validate the role of the MGAT4B gene in glioma proliferation. Briefly, 2 × 10^3^ cells were inoculated per well in 96-well plates, with 3 replicate wells set in each group. After silencing MGAT4B (SIGS0009921-1, RiboBio, China) for 22 h,34 h, and 46 h, 10 *μ*L of CCK-8 solution was added to each well and then incubated for 2 hours in a dark environment. Finally, the absorbances were analyzed under a microplate reader (BD Biosciences, USA) at 450 nm.

The migration capability of glioma cells was assessed by transwell assay and wound healing assay. Transwell assays were performed with transwell migration champers (8 *μ*m size; Corning, USA). Firstly, 5 × 10^4^ cells were uniformly planted in each upper cell chamber. As an attractant in the lower cell chamber, 500 *μ*L of DMEM culture medium with 20% fetal bovine serum (FBS) was placed. After 24 h incubation, sequentially add 4% paraformaldehyde and 0.1% crystal violet to fix and stain the cells invaded from the upper chamber. Finally, the invasive cells were captured by a CK-53 microscope and counted by the software ImageJ (Fiji version). The wound healing assay was performed on a 6-well plate. After the cells were evenly spread across the 6-well plate, the tip of a 200 *μ*L pipette was used to gently stroke vertically across the cell monolayer. The cells were subsequently cultured in DMEM medium without FBS for 24 h at 37°C. Prescratch and 24 h postscratch photographs were taken under the microscope. The migrating cells' horizontal distance away from the edge of the wound was measured with the software ImageJ (Fiji version, NIH).

The migration capability of macrophages was assessed by transwell assay. Briefly, 5 × 10^4^ THP1 cells were firstly inoculated in the upper cell chamber and induced into macrophages with PMA (200 ng/mL for 2 days). A 500 *μ*L DMEM medium that contained 5 × 10^4^ U87 or U251 cells was used as an attractant by setting it in the lower cell chamber. After 24 h incubation, sequentially add 4% paraformaldehyde and 0.1% crystal violet to fix and stain the cells invaded from the upper chamber. Finally, the invasive cells were captured by a microscope.

### 2.10. Statistical Analysis

The K-M curves were adopted to compare the difference in survival between the cohorts at risk. Cox regression and LASSO regression analyses were performed for screening out independent prognostic variants. Assessment of the diagnostic value of glycosyltransferase signature was conducted through the ROC curve analysis. The independent *t*-test was applied to compare whether there was a significant difference between the two quantitative factors. All of the statistical analyses were performed with the R program (V4.03) and GraphPad Prism 9.0. The *p* values < 0.05 was considered statistically significant.

## 3. Results

### 3.1. Consensus Clustering Analysis Elucidates the Potential Cytobiological Roles of GT Genes

**Figure 1:**
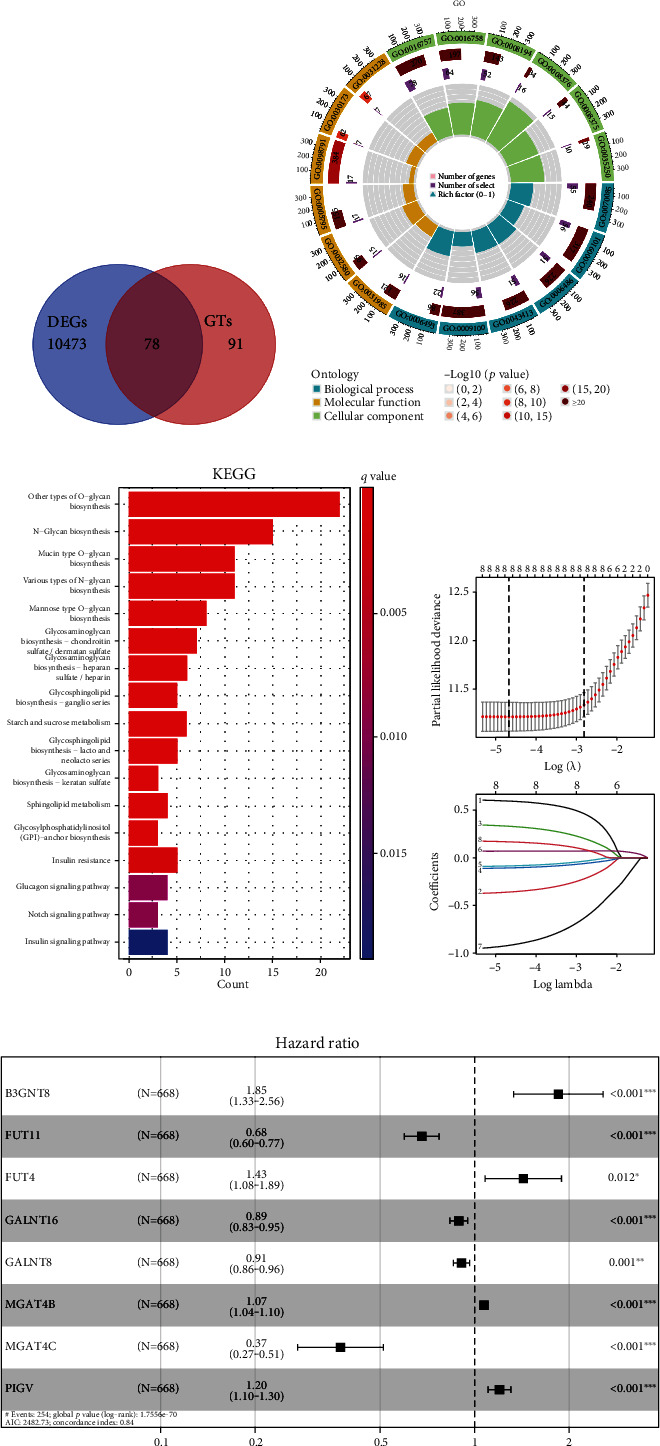
Functional enrichment analysis based on glycosyltransferase (GT) genes and construction of glycosyltransferase signature. Intersection of GT genes and differential expressed genes (a). GT DEG-related functional annotation was determined by GO (b) and KEGG (c) pathway analyses. Employing LASSO regression analysis, 8 GT genes were screened according to the optimal lambda value to be involved in the model construction (d). Multivariate Cox regression analysis was performed to screen 8 glycosyltransferase genes to construct the glycosyltransferase signature risk model (e). GT: glycosyltransferase; DEGs: differently expressed genes; GO: Gene Ontology; KEGG: Kyoto Encyclopedia of Genes and Genomes. ^∗^*p* < 0.05, ^∗∗^*p* < 0.01, and ^∗∗∗^*p* < 0.001.

The overall workflow chart was displayed in Figure S1. The potentially cytobiological roles of differentially expressed genes (DEGs) associated with GTs (Figure 1(a) and Figure S2) were revealed utilizing the GO and KEGG pathway analyses. The biological processes (BP), cellular components (CC), and molecular functions (MF) of GT genes are depicted in Figure 1(b) for the top 6 enriched GO terms. The GO enrichment terms were engaged in glycosylation, glycoprotein biosynthetic process, macromolecule glycosylation, and glycoprotein metabolic process. From the KEGG analysis, GT genes were found to be abundant in glycosaminoglycan biosynthesis, N-glycan biosynthesis, and O-glycan biosynthesis displayed in Figure 1(c).

### 3.2. Generation and Test of Glycosyltransferase Signature

Via univariate Cox regression analysis, we found that 149 of the differentially expressed GT genes were correlated with the prognosis of glioma (*p* < 0.05). Subsequently, we adopted LASSO regression to minimize the gene numbers involved in the model generation to avoid overfitting (Figure 1(d)). Then, 8 GT genes were selected to establish the glycosyltransferase signature after multivariate Cox regression analysis, based on the TCGA-based training set of 668 patients (Figure 1(f)). The formula below was applied to compute each patient's risk score:

(1) $$\begin{array}{c} \text{Risk score}=B3GNT8*0.6138-FUT11*0.3880+FUT4*0.3570-GALNT16*0.1169-GALNT8*0.0964+MGAT4B*0.0687-\text{MGAT}4\text{C}*0.9837+\text{PIGV}*0.1820. \end{array}$$

**Figure 2:**
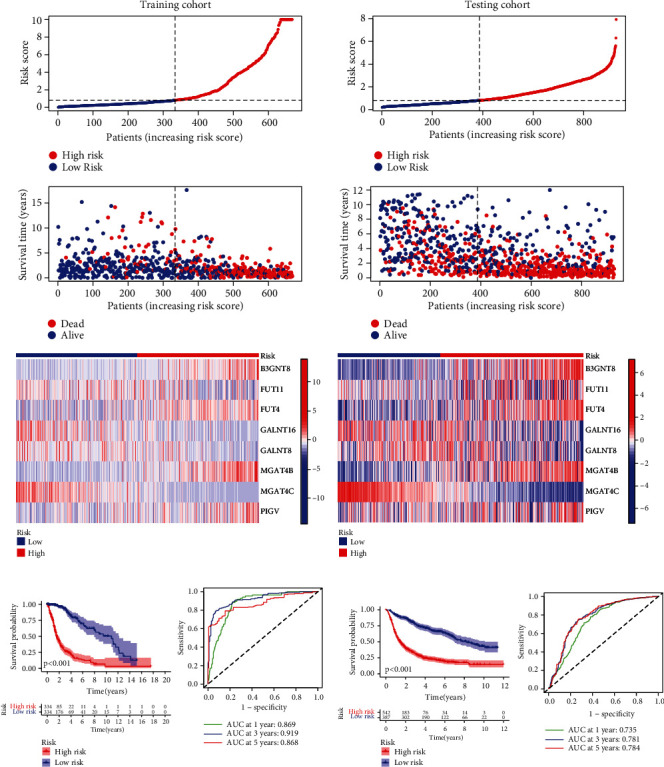
Glycosyltransferase (GT) gene expression levels and prognosis of the high- and low-risk groups in training and validation cohorts. Distribution of risk plots, survival status, and expression level of GT genes in the high- and low-risk groups in the testing group (a) and validation group (b). ROC predictive curves at 1, 3, and 5 years and K-M curve of overall survival classified by the high- and low-risk groups in training cohort (c) and validation cohort (d). GT: glycosyltransferase; K-M: Kaplan-Meier; ROC: receiver operating characteristic.

Glioma cases belonging to the TCGA training cohort were categorized into two risk groups including the low- and high-risk groups depending on the risk scores. A significantly higher percentage of patients in the high-risk group were in the fatal status compared to the low-risk group (*p* < 0.05) (Figure 2(a)). The 8 screened GT genes were also presented in heat map form for their expression levels (Figure 2(a)). Patients in the CGGA database testing cohort were categorized into low- and high-risk groups based on glycosyltransferase signature. Likewise, we obtained a similar conclusion for the testing cohort (Figure 2(b)). Findings from the K-M survival curve showed markedly shorter OS for the high-risk group in the training set, which was in accordance with the results of the test cohort (*p* < 0.05). Similarly, glycosyltransferase signature could determine patient survival with relative accuracy at 1-, 3-, and 5-year follow-ups (Figures 2(c) and 2(d)). WGCNA also found that our screened GT genes were intimately correlated with glioma survival (Figure S3).

### 3.3. Glycosyltransferase Signature Value in Glioma Patients Prognosis

**Figure 3:**
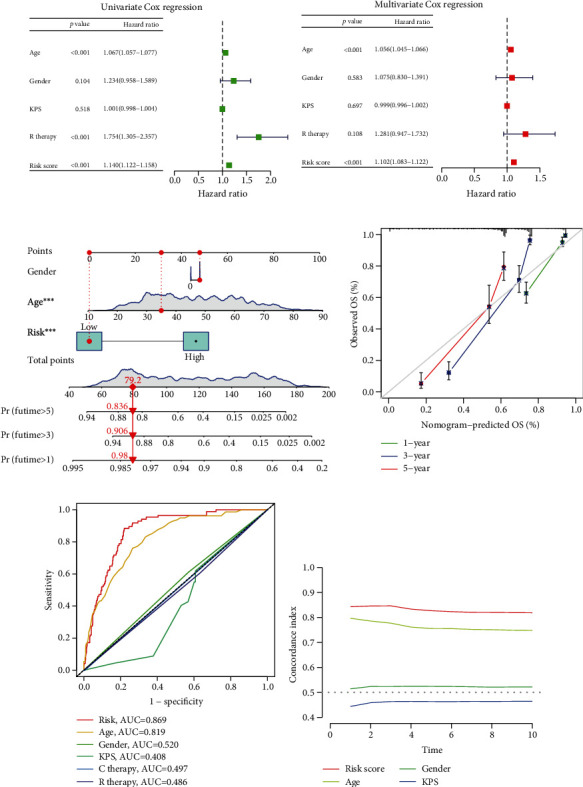
Prognostic value of the glycosyltransferase signature in training cohort. Univariate Cox regression analysis (a) and multivariate Cox regression analysis (b) were performed to compare glycosyltransferase signature risk score with other clinical factors in terms of prognostic predictive capacity. (c) Nomogram was constructed based on a combination of risk scores and other clinical factors to predict patients' 1-, 3-, and 5-year OS in a visual manner. (d) Calibration curves were utilized to compare the consistency between the predictive results and the actual 1-, 3-, and 5-year survival outcomes of glioma patients. (e) Using ROC curve analysis to compare the predictive capability of the risk model with other clinical factors. (f) Comparison of the predictive accuracy of the risk model with other clinical factors by the C-index analysis. OS: overall survival; ROC: receiver operating characteristic; AUC: area under the curve. ^∗∗∗^*p* < 0.001.

An analysis of univariate and multivariate COX regression based on TCGA and CGGA datasets showed a statistically significant correlation between prognosis and risk score (*p* < 0.001) (Figures 3(a) and 3(b) and Figure S4A-B), indicating that the glycosyltransferase signature could act as an independent and valuable prognostic indicator of glioma. To explicitly and accurately predict glioma patients' survival at 1, 3, and 5 years, we constructed a nomogram for presenting the risk scores and clinical-pathological factors (Figure 3(c) and Figure S4C). Correction curves were used to adjudicate the accuracy of this nomogram, revealing the high consistency of this nomogram with respect to the actual survival rate (Figure 3(d) and Figure S4D). Next, ROC curves at 1 year suggested that the risk score of signature had a superior prediction capacity than other factors, including age, gender, KPS, chemotherapy, and radiotherapy (Figure 3(e)). Similarly, ROC curves were mapped for 3 years based on the CGGA database to compare, and it was also found that the risk score of signature possessed a better predictive ability (Figure S4E). In addition, by calculating the C-index, risk scores were found to have higher predictive accuracy compared to several clinical factors in the TCGA database and CGGA database (Figure 3(f) and Figure S4F).

### 3.4. Comparison of Previously Reported Risk Models

**Figure 4:**
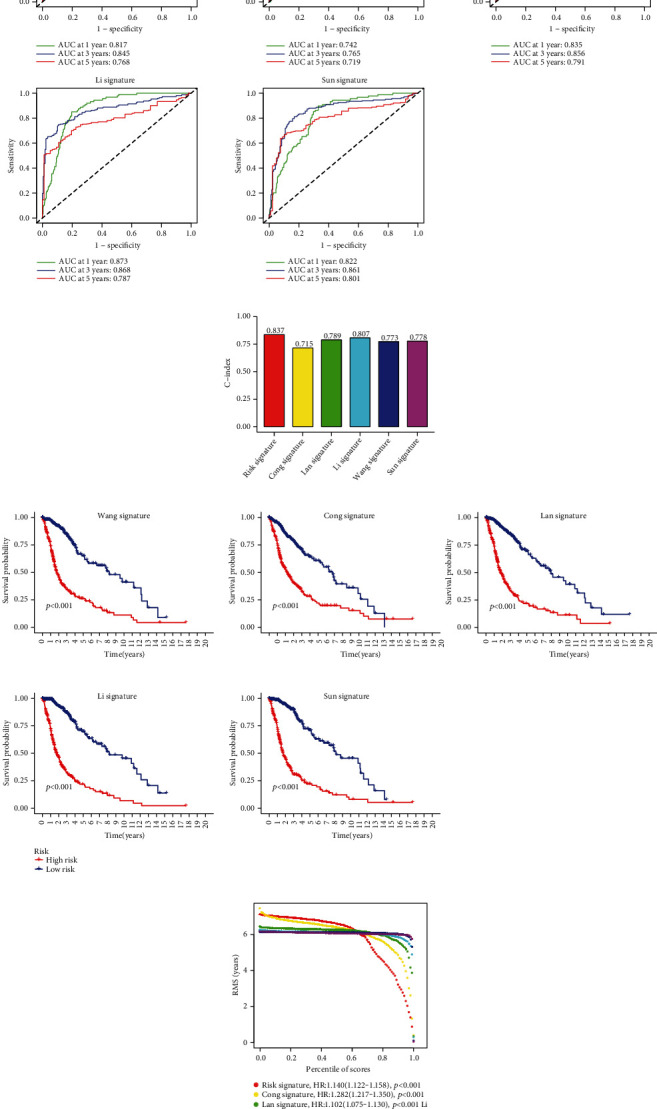
Comparison of our risk model with other risk models. (a) The ROC curves for the Wang, Cong, Lan, Li, and Sun risk models at 1, 3, and 5 years of OS. (b) C-index analysis comparison of our prognostic risk models with other 5 risk models. (c) K-M curves of the low- and high-risk groups for the Wang, Cong, Lan, Li, and Sun risk models. (d) Restricted mean survival (RMS) curves for the comparison between our risk models and other five risk models. ROC: receiver operating characteristic; KM: Kaplan-Meier.

Subsequently, we selected 5 previously published prognostic risk models for glioma to compare with our glycosyltransferase signature and correspondingly plotted ROC (Figure 4(a)). By comparing different ROC curves, our glycosyltransferase signature had higher AUC values at 3 and 5 years than these reported models. Subsequently, to compare the accuracy of different prognostic risk models, C-index was calculated with the R package “rms.” As a result, our glycosyltransferase signature possessed the largest C-index, demonstrating that the performance of our model was the best in all six prognostic models. The results showed that our glycosyltransferase signature had the highest C-index, indicating that our signature outperforms the other 5 prognostic risk signatures (Figure 4(b)). The K-M curves for the 5 models are presented in Figure 4(c). HR of the 6 models and their *p* values were acquired by using the restricted mean survival time (RMST) calculation method and are shown in Figure 4(d). RMST area and HR values indicated that our model outperforms the four models other than the Cong's, indicating the outstanding predictive capacity of our glycosyltransferase signature.

### 3.5. Clinical Relevance of Glycosyltransferase Signature

**Figure 5:**
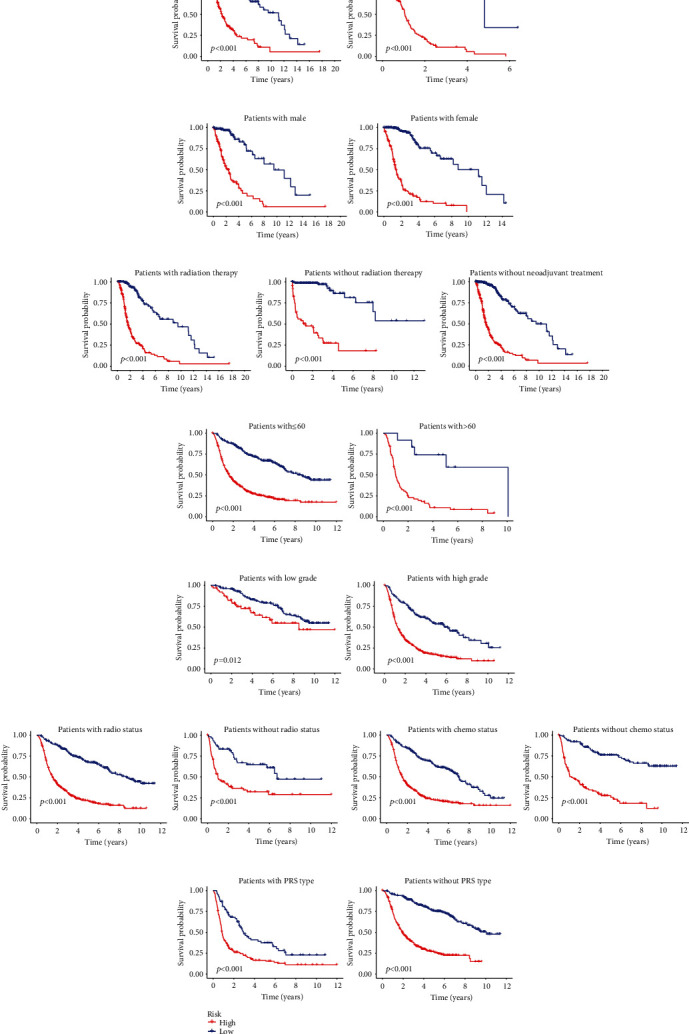
Clinical relevance of glycosyltransferase signature. (a) K-M curves to assess prognostic differences between the high- and low risk-groups in the aged and nonaged patients belonging to training cohort. (b) K-M curves to evaluate prognostic differences between the high- and low-risk groups of male and female patients belonging to the training cohort. (c) K-M curves to estimate the prognostic differences between the high- and low-risk groups of radiotherapy and nonradiotherapy patients belonging to the training cohort. (d) K-M curves to assess the prognostic differences between the high-risk and low-risk groups of patients without neoadjuvant chemotherapy in training cohort. (e) K-M curves to assess prognostic differences between the high- and low-risk groups in the aged and nonaged patients belonging to validation cohort. (f) K-M curves to evaluate prognostic differences between the high- and low-risk groups of low- and high-grade patients belonging in the validation cohort. (g) K-M curves to estimate the prognostic differences between the high- and low-risk groups of radiotherapy and nonradiotherapy patients belonging to the training cohort. (h) K-M curves to assess the prognostic differences between the high-risk and low-risk groups of patients with or without chemotherapy in training cohort. (i) K-M curves to estimate the prognostic differences between the high- and low-risk groups of PRS type and without PRS type patients in the training cohort. K-M: Kaplan-Meier; PRS: primary recurrent secondary.

After grouping by different clinical-pathological characteristics, individuals were classified into two risk groups including the low- and high-risk groups depending on their risk scores. By plotting survival K-M curves, we identified that the signature was also applied to survival prediction among the subcategories of age (Figure 5(a)), gender (Figure 5(b)), radiotherapy (Figure 5(c)), and adjuvant therapy (Figure 5(d)) in the TCGA database. By the same method, we also obtained similar results in different subcategories including age (Figure 5(e)), grade (Figure 5(f)), radiotherapy (Figure 5(g)), chemotherapy (Figure 5(h)), and PRS type (Figure 5(i)) in the CGGA database, demonstrating a strong association between risk characteristics and individual clinicopathological features. WGCNA also validated the intensive correlation between GT genes and clinical-pathological characteristics (Figure S1).

### 3.6. Glycosyltransferase Signature as a Predictor of TAM Infiltration, Immune Response, and Checkpoint Expression Levels

**Figure 6:**
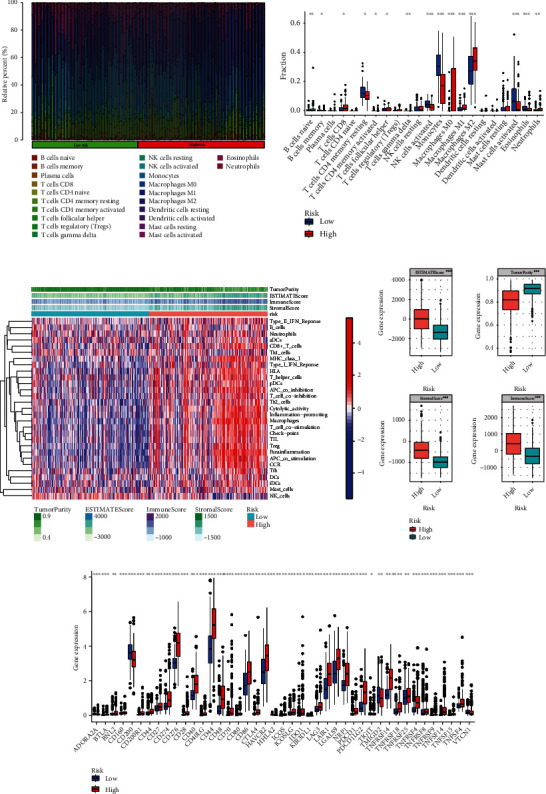
Correlation between the risk model and immune microenvironment. (a) Revealing the proportion of the 22 different immune lymphocytes among the high- and low-risk groups with Cibersort methods. (b) The fraction of the 22 different lymphocytes in the high- and low-risk groups was displayed by ssGSEA. (c) 29 immune-related pathway responses in the high- and low-risk groups. (d) ESTIMATE score analysis of the high- and low-risk groups. (e) Expression levels of immune checkpoint genes in the high- and low-risk group species. ssGSEA: single-sample gene set enrichment analysis; ESTIMATE: estimation of stromal and immune cells in malignant tumor tissues using expression data. ^∗^*p* < 0.05, ^∗∗^*p* < 0.01, and ^∗∗∗^*p* < 0.001.

The Cibersort and ssGSEA were employed to compare the proportion of the 22 different lymphocytes from the TCGA database in the high- and low-risk groups (Figures 6(a) and 6(b)). A significantly lower proportion of infiltrating macrophages was occupied by the low-risk group compared to the high-risk group (Figures 6(a)–6(b)). Infiltration of M2 TAMs was markedly high among high-risk individuals (Figures 6(a) and 6(b)). 29 immune-related pathways were quantitatively characterized by the ssGSEA approach to estimate the level of immune response enrichment in both the high- and low-risk groups (Figure 6(c)). The outcomes of the enrichment analysis revealed that immune-related pathways other than NK cell-related pathways showed dramatic enrichment within the high-risk group, especially in the activation pathway of macrophages (Figure 6(c)). The ESTIMATE algorithm validated that the ESTIMATE score, stromal score, and immune score were comparably high, whereas the high-risk group presented with lower purity of tumor (Figure 6(d)). Subsequently, a comparative analysis of gene expression levels of the immune check points (ICIs) showed that some popular ICIs, such as CTLA4, IDO1, CD274 (PD-L1), LAG3, and PDCD1 (PD-1), were found to have high expression level among the high-risk group (Figure 6(e)). Furthermore, the expression level of CD44, which partially activated PD-L1 transcription increased dramatically (Figure 6(e)).

### 3.7. Validating the Predictive Ability of the Glycosyltransferase Signature in External Clinical Cohorts

**Figure 7:**
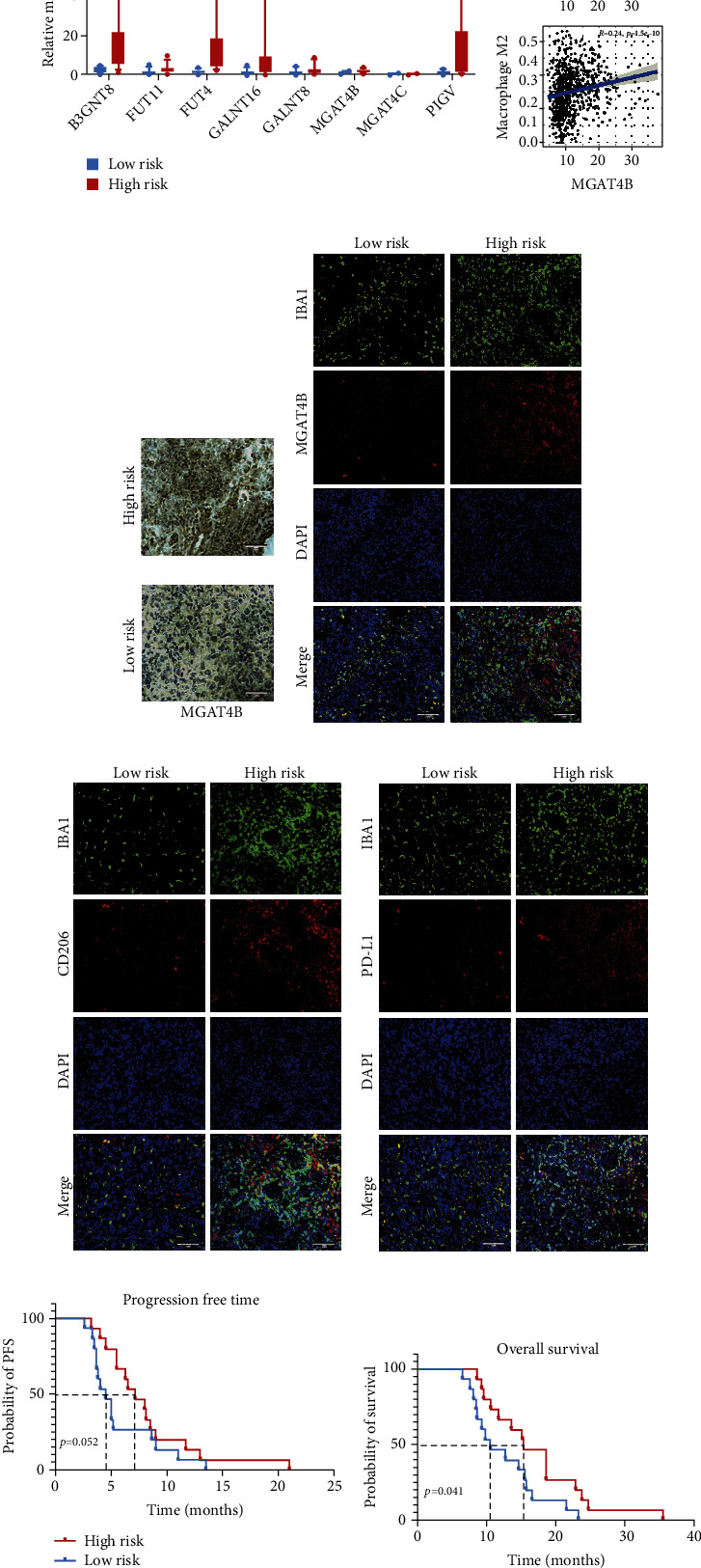
Validation of risk models in clinical cohort. (a) Expression levels of 8 risk model glycosyltransferase genes for the high-risk and low-risk groups in our clinical cohort. (b) Correlation of infiltrating macrophages with MGAT4B in the TCGA database. (c) IHC revealed elevated MGAT4B expression in the high-risk group. (d) IF costaining of MGAT4B and macrophages. (e) IF costaining of M2 marker CD206 and macrophages. (f) IF costaining of PD-L1 and macrophages. (g) Validation of the difference in progression-free time between the high- and low-risk groups in our clinical cohort with K-M curve. (h) Validation of the difference in overall survival time between the high- and low-risk groups in our clinical cohort with K-M curve. IHC: immunohistochemistry; IF: immunofluorescence; PD-L1: programmed death-ligand 1; K-M: Kaplan-Meier. ^∗^*p* < 0.05 and ^∗∗∗^*p* < 0.001.

To further validate the correlation between the glycosyltransferase signature risk model and patient survival as well as immune cell infiltration in our cohort, a total of 30 glioma patients of different grades with follow-up results were included. The 8 selected GT genes, whose expression levels were determined by qRT-PCR, were utilized to compute risk scores for 30 glioma patients (Figure 7(a)). Using the calculated risk scores, 30 glioma patients from the external cohort were assigned to the high-risk and low-risk groups for immunological and prognostic validation. MGAT4B lacked relevant evidence reported and was screened for validation from 8 genes. The MGAT4B expression level was found to be positively correlated with the infiltration of M0 macrophages and M2 macrophages, as calculated from TCGA data (Figure 7(b)). Importantly, IHC and IF validated that MGAT4B exhibited higher abundance in the tumor tissue of the high-risk group (Figures 7(c)–7(f)). Verified by our external cohort, the MGAT4B expression level in patients showed a positive correlation with the level of macrophage infiltration (Figure 7(d)), M2 macrophage infiltration (Figure 7(e)), and PD-L1 expression level (Figure 7(f)). In the prognostic analysis of the external cohort, patients among the high-risk group were identified as having shorter PFS and OS compared to the low-risk group (Figures 7(g) and 7(h)). Taken together, we validated the predictive abilities of the glycosyltransferase signature for prognosis and immunity in an external clinical cohort with our data.

### 3.8. The In Vitro Validation of MGAT4B Role in Tumor Cells

**Figure 8:**
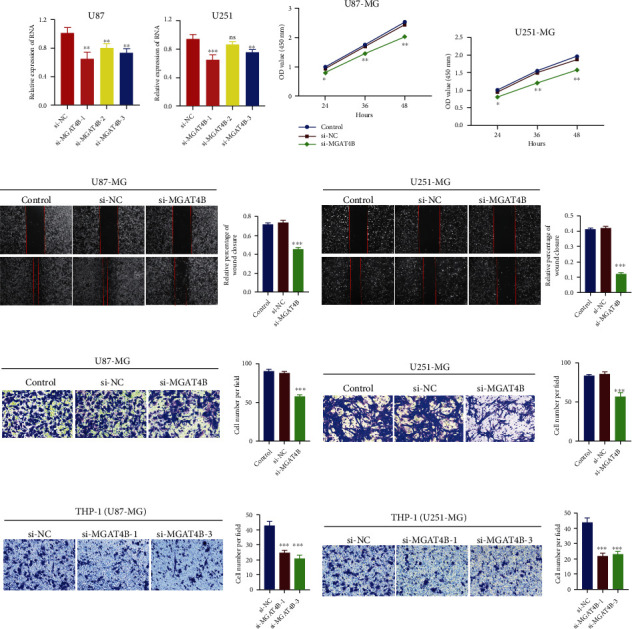
Risk model gene MGAT4B promoted glioma proliferation, invasion, and macrophage infiltration. (a) MGAT4B knockdown in U87 cell line. (b) MGAT4B knockdown in U251 cell line. CCK-8 assay was applied to estimate the proliferation capacity of U87 (c) and U251 (d) after silencing MGAT4B. Wound healing assays were performed to assess the migration ability of U87 (e) and U251 (f) cells after silencing of MGAT4B. Transwell assays were performed to assess the invasion capability of U87 (g) and U251 (h) cells after silencing MGAT4B. Transwell assay was utilized to evaluate the attraction capacity of U87 (i) and U251 (j) cells to macrophages after silencing of MGAT4B. ^∗^*p* < 0.05, ^∗∗^*p* < 0.01, and ^∗∗∗^*p* < 0.001.

By silencing the MGAT4B gene in glioma cell lines U87 and U251 (Figures 8(a) and 8(b)), we proved that MGAT4B attenuated the proliferative capacity of glioma cell lines, by using the CCK8 assay (Figures 8(c) and 8(d)). Transwell assay as well as wound healing assay revealed that the migration capacity of U87 and U251 glioma cell lines was suppressed under MGAT4B silence (Figures 8(e)–8(h)). Besides, the results of transwell experiments indicated a reduction of THP-1-derived macrophage migration ability when the MGAT4B gene of cocultured U87 and U251 cell lines was silenced (Figures 8(i) and 8(j)). Therefore, we here deciphered the promoting role of MGAT4B in glioma growth, invasion, and macrophage recruitment.

## 4. Discussion

Aberrant glycosylation expression patterns are inextricably linked to the full course of tumor development, prognosis, and drug resistance. The risk model based on GT genes provides a comprehensive scoring delineation for the prognosis and management evaluation of glioma. Here, in our research, a glycosyltransferase signature has been successfully established based on 8 GT genes, including B3GNT8, FUT11, FUT4, GALNT16, GALNT8, MGAT4B, MGAT4C, and PIGV. In addition, we confirmed that the OS was lower and the immunosuppressive response was stronger among the high-risk group as compared to the low-risk group.

Timely and accurate prognostic evaluation is a key prerequisite for the treatment of patients with glioma. These risk models, signatures, and profiles of specific genes might arouse novel strategies for exploring risk scoring and disease management tools [16]. These subjects in the risk model study may include different modes of death (iron death, apoptosis, and necroptosis), m6A modifications, epigenetic modifications (miRNA, lncRNA, and circRNA), and even some key proteins and small molecules [17]. GT genes are a class of model-building tools that deserve focused attention but have not yet been studied much. For example, Xu et al. constructed a signature encompassing immunoglobulin, GT genes, and antiviral-related genes to classify breast cancer (BC) patients into different risk groups [18]. This signature poses a robust efficacy as an independent biomarker for prognosis prediction, immunotherapy, and drug response in BC patients with high and low risk. In addition, Huang et al. also established a serum fingerprint of N-glycan, which was beneficial in discriminating intrahepatic cholangiocarcinoma (ICC) versus hepatocellular carcinoma [19]. This fingerprint also helped to aid in determining the prognosis of high- and low-risk ICC patients. Here, our study used 8 GT genes to construct the model, which has a good classification ability for the high- and low-risk patients and a higher accuracy than a single evaluation index commonly used in clinical practice. Moreover, our risk scores were capable of revealing discrepancies in immune infiltration between groups, as well as the expression levels of the immune checkpoints. Consequently, our model could predict the prognosis of glioma while providing insight into the direction of immunotherapy.

Glioma is a highly variable entity with molecular heterogeneity [20]. The activity and status of immune cells are closely tied to the course of glioma [21]. In high-grade glioma species, the immune killing effects are often modulated by the tumor microenvironment (TME) and become debilitating [22]. As a result of tumor mutations, new antigens are produced, and the exposure of these antigens may provide new targeting opportunities for immunotherapy. For instance, Zhao et al. developed a model incorporating 4 GT genes, ALG8, DCTN4, DCTN6, and UBB, to predict ovarian cancer (OC) patients and immune function [23]. The results showed that high-risk patients were at higher risk compared to low-risk patients, and tumor purity together with tumor mutational load was negatively correlated with risk scores. Our previous study also constructed a reliable signature according to the screened 9 GT genes for judging the prognosis among BC patients, which was accompanied by the excellent predictive potential regarding prognosis and immunotherapy evaluation [24]. In the present study, compared to the group at low risk, the immune status of the high-risk group showed a protumor state, accompanied by an increase in the proportion and function of specific negative immunomodulatory cells, while the proportion and activity of immunocytes with killer function were suppressed. Furthermore, high-risk patients probably tend to benefit more from treatment with ICI, resulting in longer survival. These provide novel avenues for the clinical practice of glioma treatment.

MGAT4B belongs to the N-glycan branching/antennary genes but is currently understudied and therefore included in our biological experimental validation. MGAT4B is a key gene involved in the formation of complexes between nucleotide sugar transport proteins and GTs [25]. Our study confirmed that MGAT4B was highly expressed in high-risk patient samples, implying that MGAT4B might be a biomarker that promotes tumorigenesis and progression. Furthermore, the markers of M2 macrophage CD206, together with MGAT4B, have a synergistic elevated expression profile, showing that a high MGAT4B expression state may be accompanied by a stronger immune concordance profile. Additionally, the immunoreactive microglia labeled by ionized calcium-binding adaptor molecule 1 (IBA-1) are widely dispersed in the spinal cord and tumor blocks [26]. Our study also found that the PD-L1 expression increased with the elevation of the IBA-1 expression level. These results suggest that MGAT4B is a tumor-promoting factor and is closely associated with a tumor-suppressive immune state. As for other GT genes belonging to our model, B3GNT8 was associated with malignancy and metastasis of glioma [27], while FUT11 [28], FUT4 [29], GALNT16 [30], GALNT8 [31], MGAT4C [32], and PIGV [33] have been individually studied in various type of tumors previously and are correlated with tumor malignancy, proliferation, cancer stem cells, and immune cell attraction.

TAMs are the predominant component of immune cells in brain tumors, accounting for 40% of the tumor mass [34]. Upon activation, TAMs are typically classified into two distinct phenotypes based on their functional role: proinflammatory M1 phenotype/differentiation and anti-inflammatory M2 phenotype/differentiation. A recent single-cell sequencing meta-analysis study found regional distribution differences in TAMs, with increased activity of the PD-1 signaling pathway in the tumor core and more M2 phenotype in the tumor periphery [35]. This was also supported by the evidence in our study, where we hypothesized that PD-1 signaling activation in the tumor core may be caused by elevated expression of PD-L1. As reported, PD-L1 was frequently expressed on both tumor cells as well as on M2 macrophages [36], which was also confirmed in our results. Our findings suggested that the glycosyltransferase signature was associated with increased infiltration of M2 TAMs and that signature genes could promote macrophage recruitment by tumor cells. CD44, which in part activates PD-L1 transcription employing its cleaved intracytoplasmic structural domain, was sharply elevated in high-risk patients and thus leads to antitumor immunity suppression [37]. Therefore, we argue that genotyping by glycosyltransferase signature may be beneficial in regulating therapeutic strategies against TAMs such as anti-CSF1R treatment [38].

Nevertheless, there are still several issues that need to be addressed in our study. First of all, most of our findings and conclusions are based on existing databases for exploration. Although a certain sample of external studies was also employed to conduct the reexamination, the number is still relatively small. Therefore, more and larger real-world clinical information is still essential for the efficient validation of the model. Secondly, since the number of genes has a significant impact on the accuracy of the model, it will be very interesting to consider whether other key genes need to be included. Thirdly, although we have preliminarily validated the expression and partial biological function of MGAT4B in clinical samples, other genes relevant to the construction of this glycosyltransferase signature have not been performed. The in-depth interpretation and understanding of these genes will help to more accurately assess the prognosis and immune status of glioma patients.

## 5. Conclusion

In summary, we identified a glycosyltransferase signature from the TCGA and CGGA databases as precision marker of glioma survival and TAM phenotype. We confirmed that patients belonging to the high-risk group had a poor prognosis, M2 TAM infiltration, and protumor immune pathway activation. Furthermore, high-risk genes in this signature were shown to promote glioma proliferation, invasion, and macrophage recruitment. Therefore, a comprehensive assessment of glycosylation characteristics determined by our glycosyltransferase signature helps us evaluate subsequent macrophage-targeted therapies as well as other immune microenvironment modulation therapeutic strategies.

## Data Availability

The original contributions presented in the study are included in the article/supplementary material; further inquiries can be directed to the corresponding author.

## Abbreviations

CNS: Central nervous system
WHO: World Health Organization
IDH: Isocitrate dehydrogenase
PTM: Posttranslational modification
GSCs: Glioma stem cells
TAMs: Tumor-associated macrophages
GTs: Glycosyltransferases
TCGA: The Cancer Genome Atlas
CGGA: Chinese Glioma Genome Atlas
GO: Gene Ontology
KEGG: Kyoto Encyclopedia of Genes and Genomes
FDR: False discovery rate
GGDB: GlycoGene DataBase
K-M: Kaplan-Meier
ROC: Receiver operating characteristic
KPS: Karnofsky performance scale
PRS: Primary recurrent secondary
HR: Hazard ratio
C-index: Concordance index
ssGSEA: Single-sample gene set enrichment analysis
IHC: Immunohistochemistry
IF: Immunofluorescence
CCK-8: Cell Counting Kit-8
DEGs: Differentially expressed genes
BC: Breast cancer
ICC: Intrahepatic cholangiocarcinoma
HCC: Hepatocellular carcinoma
TME: Tumor microenvironment.

## Supplementary Materials

Supplementary Materials The Supplementary Material for this article can be found online. Table S1: the clinical information of external cohorts. Table S2: the primer sequences for RT-qPCR of the 8 glycosyltransferase genes. Figure S1: the working flow chart. Figure S2: differential expressed glycosyltransferase genes. Figure S3: WGCNA to identify significant modules of glioma. (A) The determination of soft-thresholding parameter. (B) Creating a recognition coexpression network to identify modules with a cutoff value = 0.25. (C) The correlation of different modules with clinical characteristics, survival, and risk scores. (D) Expression levels of GT genes in glycosyltransferase signature among the high- and low-risk groups. Distribution of GT genes in blue (E), grey (F), and turquoise (G) modules. Figure S4: prognostic value of the glycosyltransferase signature in validation cohort. Univariate Cox regression analysis (A) and multivariate Cox regression analysis (B) were performed to compare glycosyltransferase signature risk score with other clinical factors in terms of prognostic predictive capacity. (C) Nomogram was constructed based on a combination of risk scores and other clinical factors to predict patients' 1-, 3-, and 5-year OS in a visual manner. (D) Calibration curves were utilized to compare the consistency between the predictive results and the actual 1-, 3-, and 5-year survival outcomes of glioma patients. (E) Using ROC curve analysis to compare the predictive capability of the risk model with other clinical factors. (F) Comparison of the predictive accuracy of the risk model with other clinical factors by the C-index analysis. PRS: primary recurrent secondary; OS: overall survival; ROC: receiver operating characteristic; AUC: area under the curve. ^∗∗^*p* < 0.01 and ^∗∗∗^*p* < 0.001.
