# Supplementary material for: Comprehensive Analysis Identified Glycosyltransferase Signature to Predict Glioma Prognosis and TAM Phenotype
Source: Biomed Res Int. 2023 Jan 11;2023:6082635. doi: 10.1155/2023/6082635 (PMC9859707; doi:10.1155/2023/6082635)
Supplement: Supplementary Materials — The Supplementary Material for this article can be found online. Table S1: the clinical information of external cohorts. Table S2: the primer sequences for RT-qPCR of the 8 glycosyltransferase genes. Figure S1: the working flow chart. Figure S2: differential expressed glycosyltransferase genes. Figure S3: WGCNA to identify significant modules of glioma. (A) The determination of soft-thresholding parameter. (B) Creating a recognition coexpression network to identify modules with a cutoff value = 0.25. (C) The correlation of different modules with clinical characteristics, survival, and risk scores. (D) Expression levels of GT genes in glycosyltransferase signature among the high- and low-risk groups. Distribution of GT genes in blue (E), grey (F), and turquoise (G) modules. Figure S4: prognostic value of the glycosyltransferase signature in validation cohort. Univariate Cox regression analysis (A) and multivariate Cox regression analysis (B) were performed to compare glycosyltransferase signature risk score with other clinical factors in terms of prognostic predictive capacity. (C) Nomogram was constructed based on a combination of risk scores and other clinical factors to predict patients' 1-, 3-, and 5-year OS in a visual manner. (D) Calibration curves were utilized to compare the consistency between the predictive results and the actual 1-, 3-, and 5-year survival outcomes of glioma patients. (E) Using ROC curve analysis to compare the predictive capability of the risk model with other clinical factors. (F) Comparison of the predictive accuracy of the risk model with other clinical factors by the C-index analysis. PRS: primary recurrent secondary; OS: overall survival; ROC: receiver operating characteristic; AUC: area under the curve. ∗∗p < 0.01 and ∗∗∗p < 0.001. [file 6082635.f1.zip › Figure S1.pdf]

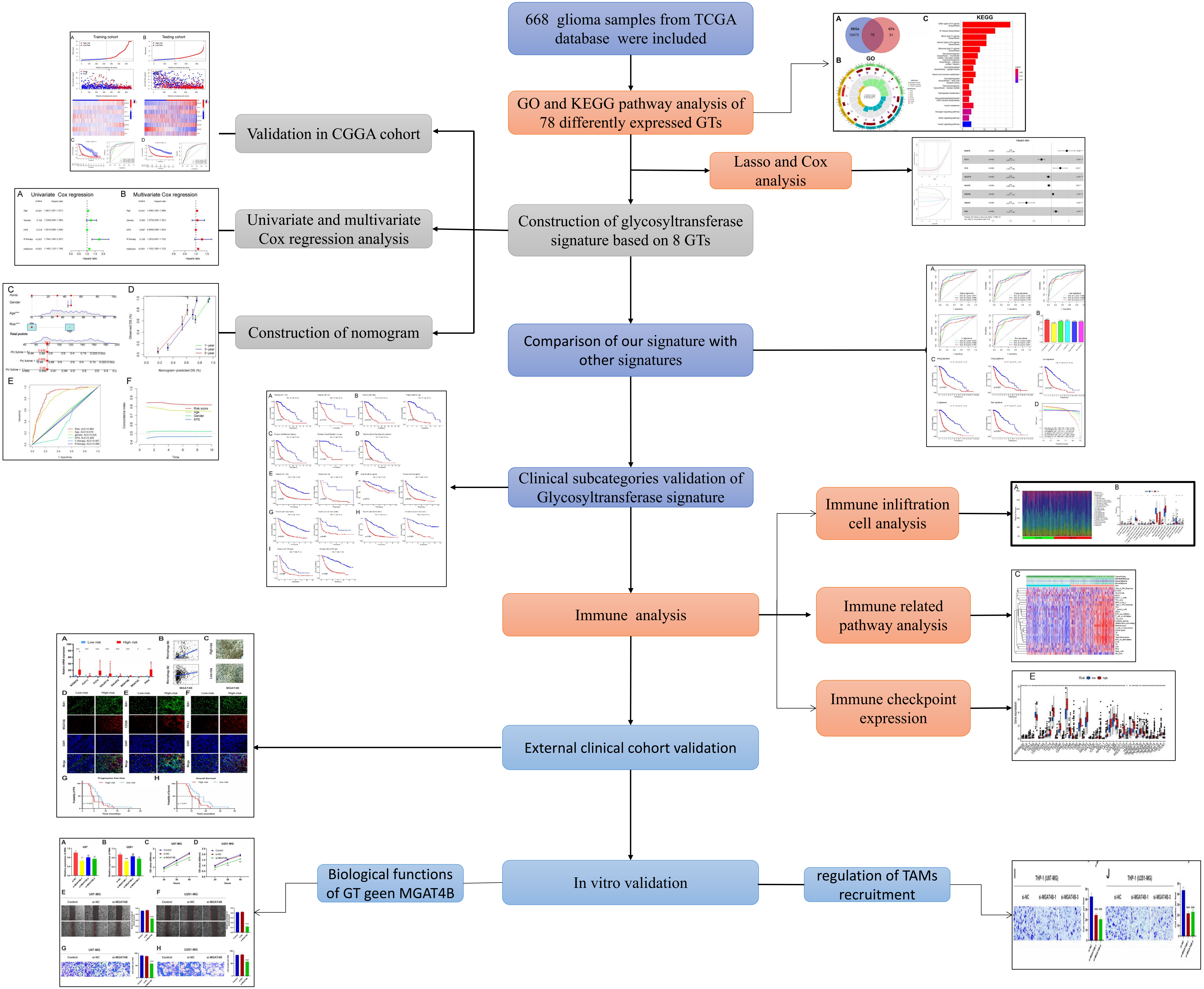

668 glioma samples from TCGA database were included
GO and KEGG pathway analysis of 78 differently expressed GTs
Lasso and Cox analysis
Construction of glycosyltransferase signature based on 8 GTs
Validation in CGGA cohort
Univariate and multivariate Cox regression analysis
Construction of nomogram
Comparison of our signature with other signatures
Clinical subcategories validation of Glycosyltransferase signature
Immune analysis
Immune inliftration cell analysis
Immune related pathway analysis
Immune checkpoint expression
External clinical cohort validation
In vitro validation
Biological functions of GT geen MGAT4B
regulation of TAMs recruitment
